# 1-(3-Carboxyl­atophen­yl)-4,4′-bipyridin-1-ium dihydrate

**DOI:** 10.1107/S1600536813024197

**Published:** 2013-09-04

**Authors:** Mengchan Fan, Zhenguo Yao, Chen Li, Zhiyong Fu

**Affiliations:** a Key Lab for Fuel Cell Technology of Guangdong Province, School of Chemistry and Chemical Engineering, South China University of Technology, Guangzhou, People’s Republic of China

## Abstract

In the crystal structure of the title compound, C_17_H_12_N_2_O_2_·2H_2_O, the carboxyl­ate group is linked *via* O—H⋯O hydrogen bonds to two water mol­ecules. The crystal packing is best described as parallel layers (viewed along the *a* axis) of viologen and water mol­ecules associated *via* O—H⋯O hydrogen bonds and π–π inter­actions, with a centroid–centroid separation of 3.8276 (9) Å.

## Related literature

For background to the applications of viologen complexes, see: Strutt *et al.* (2012 ▶). For related structures, see: Coe *et al.* (1998 ▶); Leblanc *et al.* (2010 ▶); Xu *et al.* (2007 ▶).
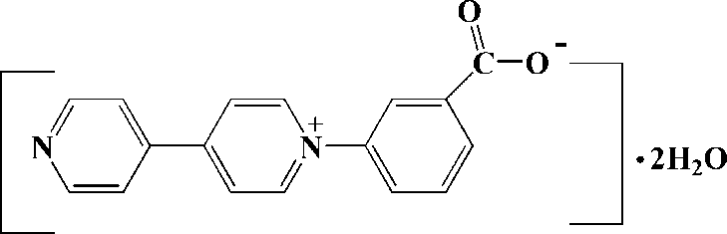


## Experimental

### 

#### Crystal data

C_17_H_12_N_2_O_2_·2H_2_O
*M* *_r_* = 312.32
Triclinic, 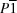

*a* = 7.8700 (16) Å
*b* = 10.090 (2) Å
*c* = 10.250 (2) Å
α = 81.36 (3)°
β = 73.13 (3)°
γ = 74.64 (3)°
*V* = 748.7 (3) Å^3^
*Z* = 2
Mo *K*α radiation
μ = 0.10 mm^−1^
*T* = 298 K
0.13 × 0.12 × 0.12 mm

#### Data collection

Bruker SMART CCD diffractometer
Absorption correction: multi-scan (*SADABS*; Bruker, 2001 ▶) *T*_min_ = 0.971, *T*_max_ = 0.993
6136 measured reflections
2741 independent reflections
2120 reflections with *I* > 2σ(*I*)
*R*_int_ = 0.014

#### Refinement

*R*[*F* ^2^ > 2σ(*F* ^2^)] = 0.042
*wR*(*F* ^2^) = 0.172
*S* = 1.18
2741 reflections
221 parameters
4 restraints
H atoms treated by a mixture of independent and constrained refinement
Δρ_max_ = 0.26 e Å^−3^
Δρ_min_ = −0.29 e Å^−3^

### 

Data collection: *SMART* (Bruker, 2001 ▶); cell refinement: *SAINT* (Bruker, 2001 ▶); data reduction: *SAINT*; program(s) used to solve structure: *SHELXS2013* (Sheldrick, 2008 ▶); program(s) used to refine structure: *SHELXL2013* (Sheldrick, 2008 ▶); molecular graphics: *SHELXTL* (Sheldrick, 2008 ▶); software used to prepare material for publication: *SHELXTL*.

## Supplementary Material

Crystal structure: contains datablock(s) global, I. DOI: 10.1107/S1600536813024197/fj2640sup1.cif

Structure factors: contains datablock(s) I. DOI: 10.1107/S1600536813024197/fj2640Isup2.hkl

Supplementary material file. DOI: 10.1107/S1600536813024197/fj2640Isup3.cml

Additional supplementary materials:  crystallographic information; 3D view; checkCIF report

## Figures and Tables

**Table 1:** Hydrogen-bond geometry (Å, °)

*D*—H⋯*A*	*D*—H	H⋯*A*	*D*⋯*A*	*D*—H⋯*A*
O4—H3⋯O1	0.89 (3)	1.84 (2)	2.703 (2)	164 (1)
O3—H1⋯O2	0.91 (2)	1.94 (2)	2.843 (3)	174 (1)

**Fig. 1.**
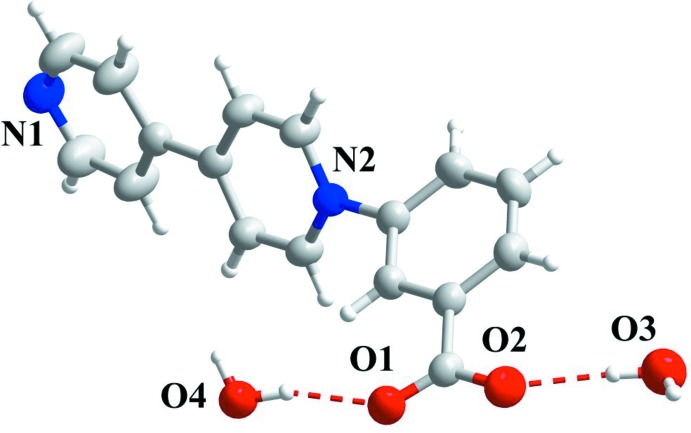
The molecular structure of (I), with atom labels and 50% probability displacement ellipsoids for non-H atoms.

**Fig. 2.**
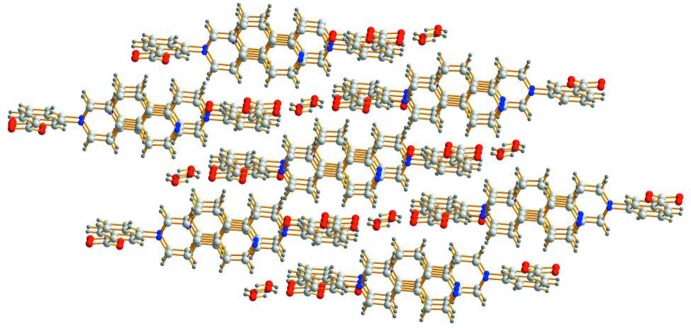
Layer structure of (I).

## supplementary crystallographic information

### 1. Comment

Viologen species are electron deficient compounds, which have been used as electron acceptor in the construction of charge transfer molecular systems and donor-acceptor-type photochromic materials (Strutt *et al.*, 2012). Recently, many classes of photosensitive model systems have been explored with dimethyl-,diethyl-, dibetaine- and benzyl viologens as ligands (Coe *et al.* 1998; Leblanc *et al.*, 2010; Xu *et al.*, 2007). Here we report the synthesis and characterization of a new viologen compound. Single crystal data indicate that the asymmetric unit of the title compound contains a monosubstituted pyridylium molecule (1-(3-carboxyphenyl)-4,4,-bipyridinium), and two water molecule (Figure 1). The carboxyl group of viologen molecule is hydrogen bonded in a O···H—O manner with two adjacent water molecules (O1···O4 = 2.7034 Å, O1···O4 = 2.8427 Å). The pyridylium molecules are arranged in a parallel packing mode. And the phenyl rings among them interact with each other through π–π stacking [centroid-centroid separation = 3.8276 (9) Å]. These noncovalent forces link the adjcent units and generate a two dimensional supramolecular network (Figure 2).

### 2. Experimental

*N*-(3-carboxyphenyl)-4,4,-bipyridinium chloride (0.33 mmol,0.092 g) and CuI(0.66 mmol, 0.13 g) were added to a solution of triethylamine (0.5 mL) and ethanol (10 mL). The solution was refluxed for 24 h. To the mixture was added 20 mL dichloromethane, yellow crystals (0.048 g, 0.17 mmol) were obtained after one day.

### 3. Refinement

H atoms of carbon were positioned geometrically and refined using a riding model, with C—H = 0.93–0.97 Å. H atoms of water molecules were located from the difference fourier map, with O—H = 0.85–0.87 Å. Non-hydrogen atoms were refined anisotropically.

Data collection: SMART (Bruker, 2001); cell refinement: SAINT (Bruker, 2001); data reduction: SAINT; program(s) used to solve structure:SHELXS 2013; program(s) used to refine structure:SHELXL 2013; molecular graphics: Bruker SHELXTL; software used to prepare material for publication: SHELXTL 2013.

### Crystal data

**Table:**

C_17_H_12_N_2_O_2_·2H_2_O	*Z* = 2
*M**_r_* = 312.32	*F*(000) = 328
Triclinic, *P*1	*D*_x_ = 1.385 Mg m^−^^3^
*a* = 7.8700 (16) Å	Mo *K*α radiation, λ = 0.71073 Å
*b* = 10.090 (2) Å	Cell parameters from 6136 reflections
*c* = 10.250 (2) Å	θ = 3.0–25.4°
α = 81.36 (3)°	µ = 0.10 mm^−^^1^
β = 73.13 (3)°	*T* = 298 K
γ = 74.64 (3)°	Block, yellow
*V* = 748.7 (3) Å^3^	0.13 × 0.12 × 0.12 mm

### Data collection

**Table:**

Bruker SMART CCD diffractometer	2120 reflections with *I* > 2σ(*I*)
Radiation source: fine-focus tube	*R*_int_ = 0.014
ω scans	θ_max_ = 25.4°, θ_min_ = 3.0°
Absorption correction: multi-scan (*SADABS*; Bruker, 2001)	*h* = −9→9
*T*_min_ = 0.971, *T*_max_ = 0.993	*k* = −12→12
6136 measured reflections	*l* = −12→12
2741 independent reflections	

### Refinement

**Table:**

Refinement on *F*^2^	4 restraints
Least-squares matrix: full	Hydrogen site location: inferred from neighbouring sites
*R*[*F*^2^ > 2σ(*F*^2^)] = 0.042	H atoms treated by a mixture of independent and constrained refinement
*wR*(*F*^2^) = 0.172	*w* = 1/[σ^2^(*F*_o_^2^) + (0.1108*P*)^2^ + 0.0207*P*] where *P* = (*F*_o_^2^ + 2*F*_c_^2^)/3
*S* = 1.18	(Δ/σ)_max_ < 0.001
2741 reflections	Δρ_max_ = 0.26 e Å^−^^3^
221 parameters	Δρ_min_ = −0.29 e Å^−^^3^

### Special details

**Table:**

Geometry. All e.s.d.'s (except the e.s.d. in the dihedral angle between two l.s. planes) are estimated using the full covariance matrix. The cell e.s.d.'s are taken into account individually in the estimation of e.s.d.'s in distances, angles and torsion angles; correlations between e.s.d.'s in cell parameters are only used when they are defined by crystal symmetry. An approximate (isotropic) treatment of cell e.s.d.'s is used for estimating e.s.d.'s involving l.s. planes.

### Fractional atomic coordinates and isotropic or equivalent isotropic displacement parameters (Å^2^)

**Table:**

	*x*	*y*	*z*	*U*_iso_*/*U*_eq_	
O1	−0.30377 (18)	0.27817 (16)	0.65560 (13)	0.0580 (4)	
O2	−0.32509 (18)	0.40158 (14)	0.82326 (13)	0.0604 (4)	
O3	−0.2973 (2)	0.5061 (2)	1.05692 (18)	0.0752 (5)	
H1	−0.297 (4)	0.472 (3)	0.980 (2)	0.113*	
H2	−0.411 (3)	0.549 (3)	1.091 (3)	0.113*	
O4	−0.2512 (2)	0.16627 (17)	0.41923 (14)	0.0644 (4)	
H3	−0.255 (4)	0.213 (3)	0.487 (2)	0.087*	
H4	−0.177 (4)	0.192 (3)	0.348 (2)	0.113 (11)*	
N1	0.9650 (2)	−0.16420 (18)	−0.16937 (16)	0.0582 (5)	
N2	0.35792 (18)	0.20963 (14)	0.37683 (13)	0.0390 (4)	
C1	0.8954 (3)	−0.2281 (2)	−0.0513 (2)	0.0700 (7)	
H1A	0.9321	−0.3238	−0.0414	0.084*	
C2	0.7715 (3)	−0.1613 (2)	0.0583 (2)	0.0640 (6)	
H2A	0.7263	−0.2118	0.1387	0.077*	
C3	0.9079 (3)	−0.0286 (2)	−0.1783 (2)	0.0677 (6)	
H3A	0.9535	0.0193	−0.2605	0.081*	
C4	0.7868 (3)	0.0465 (2)	−0.0755 (2)	0.0635 (6)	
H4A	0.7529	0.1421	−0.0888	0.076*	
C5	0.7152 (2)	−0.01950 (17)	0.04765 (16)	0.0402 (4)	
C6	0.5871 (2)	0.05920 (17)	0.16200 (16)	0.0392 (4)	
C7	0.4871 (2)	−0.00366 (18)	0.27897 (17)	0.0442 (4)	
H7A	0.4963	−0.0981	0.2854	0.053*	
C8	0.3756 (2)	0.07283 (18)	0.38421 (17)	0.0440 (4)	
H8A	0.3111	0.0294	0.4621	0.053*	
C9	0.5631 (3)	0.20167 (19)	0.15781 (18)	0.0501 (5)	
H9A	0.6250	0.2481	0.0809	0.060*	
C10	0.4506 (3)	0.27350 (18)	0.26465 (18)	0.0484 (5)	
H10A	0.4375	0.3683	0.2602	0.058*	
C11	0.2450 (2)	0.28855 (17)	0.49151 (16)	0.0388 (4)	
C12	0.3181 (2)	0.37361 (19)	0.54246 (18)	0.0469 (5)	
H12A	0.4384	0.3802	0.5042	0.056*	
C13	0.2094 (3)	0.4484 (2)	0.65104 (19)	0.0516 (5)	
H13A	0.2568	0.5055	0.6871	0.062*	
C14	0.0297 (3)	0.43901 (19)	0.70679 (18)	0.0469 (4)	
H14A	−0.0429	0.4906	0.7795	0.056*	
C15	0.0660 (2)	0.27777 (17)	0.54661 (16)	0.0393 (4)	
H15A	0.0194	0.2200	0.5108	0.047*	
C16	−0.0432 (2)	0.35330 (17)	0.65519 (15)	0.0388 (4)	
C17	−0.2405 (2)	0.34373 (17)	0.71527 (16)	0.0413 (4)	

### Atomic displacement parameters (Å^2^)

**Table:**

	*U*^11^	*U*^22^	*U*^33^	*U*^12^	*U*^13^	*U*^23^
O1	0.0458 (8)	0.0799 (10)	0.0496 (7)	−0.0242 (7)	0.0005 (6)	−0.0184 (7)
O2	0.0515 (8)	0.0625 (9)	0.0572 (8)	−0.0111 (7)	0.0101 (6)	−0.0272 (7)
O3	0.0608 (10)	0.0918 (13)	0.0733 (10)	−0.0222 (9)	−0.0073 (8)	−0.0201 (9)
O4	0.0642 (10)	0.0820 (11)	0.0501 (8)	−0.0358 (8)	0.0032 (7)	−0.0173 (7)
N1	0.0570 (10)	0.0578 (10)	0.0506 (9)	−0.0097 (8)	0.0010 (7)	−0.0132 (8)
N2	0.0331 (7)	0.0408 (8)	0.0408 (7)	−0.0077 (6)	−0.0065 (6)	−0.0044 (6)
C1	0.0842 (16)	0.0437 (11)	0.0632 (13)	−0.0089 (10)	0.0087 (11)	−0.0138 (10)
C2	0.0801 (15)	0.0448 (11)	0.0489 (11)	−0.0109 (10)	0.0079 (10)	−0.0045 (9)
C3	0.0795 (15)	0.0595 (13)	0.0436 (10)	−0.0115 (11)	0.0092 (10)	−0.0007 (9)
C4	0.0781 (14)	0.0440 (10)	0.0470 (11)	−0.0038 (9)	0.0059 (9)	−0.0017 (9)
C5	0.0384 (9)	0.0431 (9)	0.0388 (9)	−0.0099 (7)	−0.0086 (7)	−0.0042 (7)
C6	0.0371 (9)	0.0402 (9)	0.0388 (9)	−0.0085 (7)	−0.0085 (7)	−0.0023 (7)
C7	0.0495 (10)	0.0378 (9)	0.0420 (9)	−0.0125 (7)	−0.0041 (7)	−0.0041 (7)
C8	0.0431 (9)	0.0430 (9)	0.0417 (9)	−0.0132 (7)	−0.0028 (7)	−0.0013 (7)
C9	0.0538 (11)	0.0420 (10)	0.0438 (9)	−0.0116 (8)	0.0016 (8)	0.0008 (8)
C10	0.0528 (11)	0.0362 (9)	0.0472 (10)	−0.0093 (8)	−0.0011 (8)	−0.0024 (8)
C11	0.0347 (9)	0.0409 (9)	0.0379 (8)	−0.0050 (7)	−0.0074 (7)	−0.0054 (7)
C12	0.0367 (9)	0.0502 (10)	0.0546 (10)	−0.0110 (8)	−0.0101 (8)	−0.0090 (8)
C13	0.0538 (11)	0.0529 (11)	0.0551 (10)	−0.0166 (9)	−0.0162 (8)	−0.0145 (9)
C14	0.0494 (10)	0.0452 (10)	0.0434 (9)	−0.0077 (8)	−0.0076 (7)	−0.0109 (8)
C15	0.0375 (9)	0.0410 (9)	0.0396 (9)	−0.0085 (7)	−0.0087 (7)	−0.0076 (7)
C16	0.0389 (9)	0.0378 (8)	0.0361 (8)	−0.0048 (7)	−0.0077 (7)	−0.0036 (7)
C17	0.0420 (9)	0.0383 (9)	0.0379 (8)	−0.0058 (7)	−0.0035 (7)	−0.0061 (7)

### Geometric parameters (Å, º)

**Table:**

O1—C17	1.238 (2)	C8—H8A	0.9300
O2—C17	1.253 (2)	C9—C10	1.358 (3)
N1—C3	1.320 (3)	C9—H9A	0.9300
N1—C1	1.322 (3)	C10—H10A	0.9300
N2—C8	1.343 (2)	C11—C12	1.383 (3)
N2—C10	1.345 (2)	C11—C15	1.384 (2)
N2—C11	1.451 (2)	C12—C13	1.378 (3)
C1—C2	1.382 (3)	C12—H12A	0.9300
C1—H1A	0.9300	C13—C14	1.386 (3)
C2—C5	1.379 (3)	C13—H13A	0.9300
C2—H2A	0.9300	C14—C16	1.390 (3)
C3—C4	1.367 (3)	C14—H14A	0.9300
C3—H3A	0.9300	C15—C16	1.382 (2)
C4—C5	1.374 (3)	C15—H15A	0.9300
C4—H4A	0.9300	C16—C17	1.519 (2)
C5—C6	1.480 (2)	O4—H3	0.891 (17)
C6—C9	1.395 (2)	O4—H4	0.847 (17)
C6—C7	1.396 (2)	O3—H2	0.880 (18)
C7—C8	1.368 (2)	O3—H1	0.904 (18)
C7—H7A	0.9300		
			
C3—N1—C1	115.64 (17)	C10—C9—H9A	119.6
C8—N2—C10	119.98 (15)	C6—C9—H9A	119.6
C8—N2—C11	120.41 (14)	N2—C10—C9	121.01 (16)
C10—N2—C11	119.57 (14)	N2—C10—H10A	119.5
N1—C1—C2	123.91 (19)	C9—C10—H10A	119.5
N1—C1—H1A	118.0	C12—C11—C15	121.54 (16)
C2—C1—H1A	118.0	C12—C11—N2	119.25 (15)
C5—C2—C1	119.65 (17)	C15—C11—N2	119.20 (15)
C5—C2—H2A	120.2	C13—C12—C11	118.75 (16)
C1—C2—H2A	120.2	C13—C12—H12A	120.6
N1—C3—C4	124.66 (18)	C11—C12—H12A	120.6
N1—C3—H3A	117.7	C12—C13—C14	120.29 (17)
C4—C3—H3A	117.7	C12—C13—H13A	119.9
C3—C4—C5	119.83 (18)	C14—C13—H13A	119.9
C3—C4—H4A	120.1	C13—C14—C16	120.73 (17)
C5—C4—H4A	120.1	C13—C14—H14A	119.6
C4—C5—C2	116.29 (17)	C16—C14—H14A	119.6
C4—C5—C6	121.07 (16)	C16—C15—C11	119.65 (16)
C2—C5—C6	122.63 (15)	C16—C15—H15A	120.2
C9—C6—C7	116.72 (16)	C11—C15—H15A	120.2
C9—C6—C5	120.71 (15)	C15—C16—C14	119.05 (16)
C7—C6—C5	122.56 (15)	C15—C16—C17	120.11 (15)
C8—C7—C6	120.38 (16)	C14—C16—C17	120.83 (16)
C8—C7—H7A	119.8	O1—C17—O2	125.33 (17)
C6—C7—H7A	119.8	O1—C17—C16	117.90 (15)
N2—C8—C7	121.04 (15)	O2—C17—C16	116.75 (16)
N2—C8—H8A	119.5	H3—O4—H4	108 (3)
C7—C8—H8A	119.5	H2—O3—H1	105 (3)
C10—C9—C6	120.85 (16)		
			
C3—N1—C1—C2	−0.3 (4)	C11—N2—C10—C9	177.37 (16)
N1—C1—C2—C5	−0.5 (4)	C6—C9—C10—N2	−0.7 (3)
C1—N1—C3—C4	0.7 (4)	C8—N2—C11—C12	127.44 (17)
N1—C3—C4—C5	−0.2 (4)	C10—N2—C11—C12	−50.1 (2)
C3—C4—C5—C2	−0.6 (3)	C8—N2—C11—C15	−53.1 (2)
C3—C4—C5—C6	178.5 (2)	C10—N2—C11—C15	129.41 (17)
C1—C2—C5—C4	0.9 (3)	C15—C11—C12—C13	0.3 (3)
C1—C2—C5—C6	−178.2 (2)	N2—C11—C12—C13	179.77 (15)
C4—C5—C6—C9	−12.6 (3)	C11—C12—C13—C14	−0.6 (3)
C2—C5—C6—C9	166.49 (19)	C12—C13—C14—C16	0.6 (3)
C4—C5—C6—C7	168.32 (19)	C12—C11—C15—C16	−0.1 (2)
C2—C5—C6—C7	−12.6 (3)	N2—C11—C15—C16	−179.52 (13)
C9—C6—C7—C8	−1.6 (3)	C11—C15—C16—C14	0.1 (2)
C5—C6—C7—C8	177.51 (15)	C11—C15—C16—C17	179.20 (14)
C10—N2—C8—C7	0.0 (3)	C13—C14—C16—C15	−0.4 (3)
C11—N2—C8—C7	−177.48 (15)	C13—C14—C16—C17	−179.46 (15)
C6—C7—C8—N2	0.9 (3)	C15—C16—C17—O1	−7.1 (2)
C7—C6—C9—C10	1.5 (3)	C14—C16—C17—O1	171.97 (16)
C5—C6—C9—C10	−177.65 (16)	C15—C16—C17—O2	171.73 (14)
C8—N2—C10—C9	−0.1 (3)	C14—C16—C17—O2	−9.2 (2)

### Hydrogen-bond geometry (Å, º)

**Table:**

*D*—H···*A*	*D*—H	H···*A*	*D*···*A*	*D*—H···*A*
O4—H3···O1	0.89 (3)	1.84 (2)	2.703 (2)	164 (1)
O3—H1···O2	0.91 (2)	1.94 (2)	2.843 (3)	174 (1)
